# Leveling up Organic Semiconductors with Crystal Twisting

**DOI:** 10.1021/acs.cgd.3c01072

**Published:** 2023-11-14

**Authors:** St. John Whittaker, Hengyu Zhou, Rochelle B. Spencer, Yongfan Yang, Akash Tiwari, Justin Bendesky, Merritt McDowell, Pallavi Sundaram, Idalys Lozano, Shin Kim, Zhihua An, Alexander G. Shtukenberg, Bart Kahr, Stephanie S. Lee

**Affiliations:** Molecular Design Institute, Department of Chemistry, New York University, New York, New York 10003, United States

## Abstract

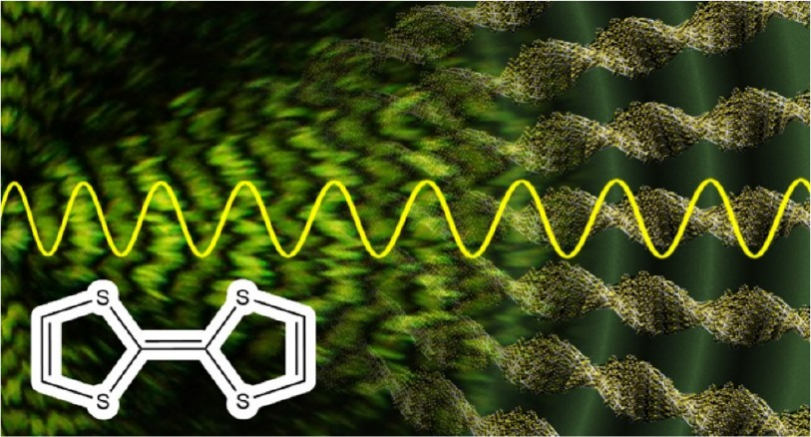

The performance of crystalline organic semiconductors depends on the solid-state structure, especially the orientation of the conjugated components with respect to device platforms. Often, crystals can be engineered by modifying chromophore substituents through synthesis. Meanwhile, dissymetry is necessary for high-tech applications like chiral sensing, optical telecommunications, and data storage. The synthesis of dissymmetric molecules is a labor-intensive exercise that might be undermined because common processing methods offer little control over orientation. Crystal twisting has emerged as a generalizable method for processing organic semiconductors and offers unique advantages, such as patterning of physical and chemical properties and chirality that arises from mesoscale twisting. The precession of crystal orientations can enrich performance because achiral molecules in achiral space groups suddenly become candidates for the aforementioned technologies that require dissymetry.

## Abstract 2

This perspective reviews recent progress in the use of spontaneous crystal twisting to embed periodic patterns of material properties, including absorbance, fluorescence, and conductivity, into organic semiconductor films. Emergent properties that will enable centrosymmetric organic semiconductors to be repurposed for chiroptoelectronics are also highlighted.

## Introduction

Since the discovery of induced electrical conductivity in anthracene crystals by Kallmann and Pope in 1960,^1^ hundreds of semiconducting molecules have been designed and incorporated into optoelectronics, from solar cells and light-emitting diodes to chemical sensors and memory storage. While organic semiconductors exhibit orders of magnitude lower charge mobilities (ca. 1 cm^2^/(V s)) compared to inorganics (ca. 1000 cm^2^/(V s)), synthetic tunability of organic material properties such as band gap, solubility, and color, coupled with the ease of processing from solution and the melt, promise applications beyond those of silicon-based devices.

Controlling crystal orientation during film processing remains a critical challenge in realizing the potential of organic semiconductors. Kallmann and Pope wrestled with anthracene anisotropy from the start.^2,3^ Optoelectronic properties, including absorptivity, photoluminescence, and charge mobility, vary along different crystallographic directions. For the best performance, the fast charge transport direction (typically the π-stack direction) should align with the direction of current flow in devices. For sandwich electrode structures, such as those used in solar cells and organic light-emitting diodes (OLEDs), this direction is perpendicular to the substrate surface. For organic field-effect transistors (OFETs) with coplanar electrodes, on the other hand, current flow is parallel to the substrate surface. In practice, however, rapid crystallization from solution or the melt during film processing generally precludes orientation control. Molecule–substrate interactions often dictate crystal orientations perpendicular to the substrate surface, limiting the use of many molecules in sandwich electrode architectures. Compound specific interactions require approaching orientation on a case-by-case basis.^4−9^

The growth of twisted crystals is a generalizable strategy to overcome such constraints in organic electronics. This spontaneous phenomenon is not limited to specific chemical structures, space groups, material classes, or deposition methods—twisting has been observed in inorganic, polymer, and small-molecule crystals grown from vapors, solutions, and melts.^172^ As crystals twist about the growth direction, all the normal crystallographic orientations present themselves perpendicular to the substrate surface, with each rotation of π radians. Anisotropy is thus patterned into the film, minimizing the need for orientation control. Twisting also imparts chirality, enabling potentially hundreds of centrosymmetric organic semiconductors designed over the past 50 years to be repurposed for chiroptoelectronics that discriminately transmit, emit, and detect circularly polarized light (CPL).

In this perspective, we review recent progress in twisted organic semiconductor crystals for optoelectronics and chiroptoelectronics. Advances broadly fall into two categories: (1) twist-induced improvements in optoelectronic properties by accessing varied out-of-plane crystal orientations and (2) emergent properties reliant on microstructure and chirality. After introducing crystal twisting, we first review orientation-dependent properties, including absorptivity, photoluminescence, conductivity, and photoconductivity that are modulated by twisting. We then discuss emergent properties that may arise because of twisting, such as iridescence, circular birefringence, and electric magnetochiral anisotropy. Finally, we discuss current limitations of twisting as a processing strategy and outline future directions.

## Background

### Brief Overview of Crystal Twisting

Spontaneous crystal twisting has been observed across material classes, including inorganic oxides,^10−21^ small organic molecules,^22−33^ and polymers,^34−49^ grown from vapors, solutions, and melts. In some cases, twisted single crystals grow in isolation,^50,51^ but twisting is more frequently observed during spherulitic growth sandwiched between glass substrates in which crystal fibers grow radially.^22^ Eventually, all in-plane directions from a crystal nucleus occur through noncrystallographic branching, such that the final polycrystalline aggregate, when pressed between glass, is disk-like. Spherulitic crystallization typically occurs under far-from-equilibrium conditions, e.g. high supercooling for crystallization from the melt.

**Figure 1:**
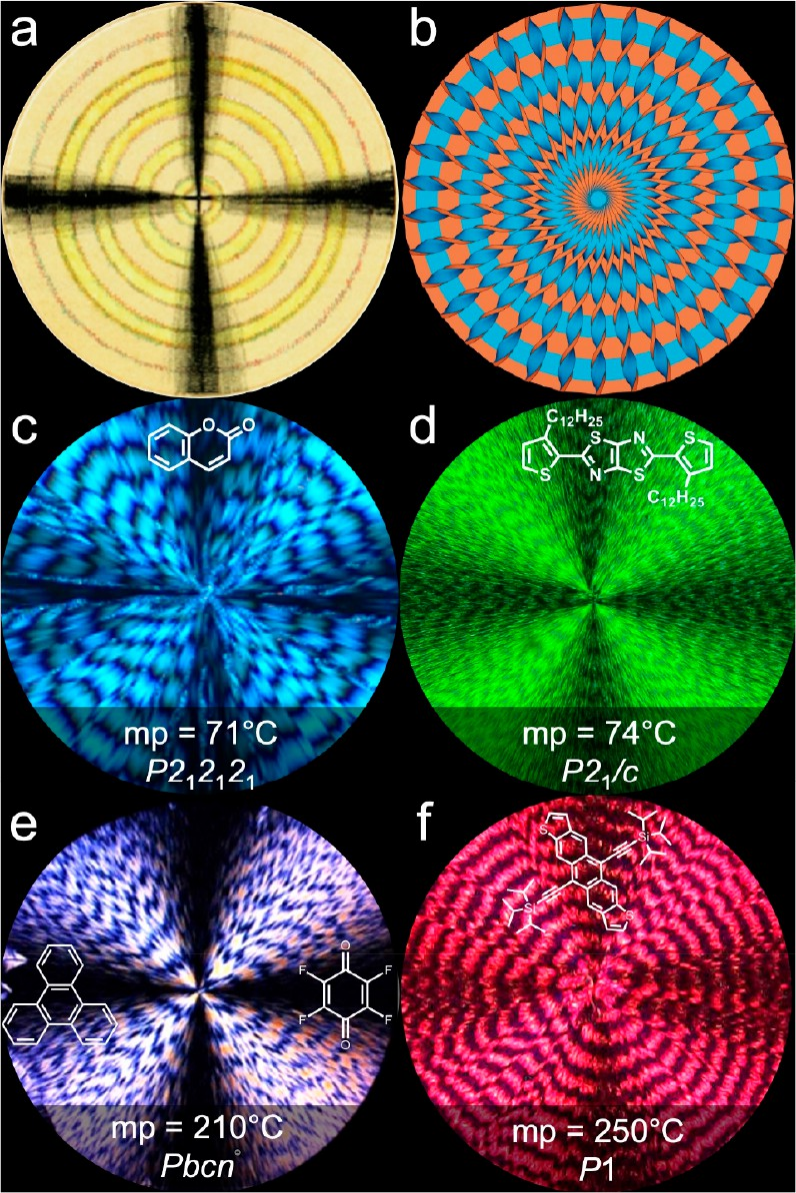
(a) Drawing of an ammonium oxalurate banded spherulite viewed between crossed polarizers by Brewster.^52,53^ (b) Illustration of a banded spherulite comprised of helicoidal fibrils emanating radially from the nucleation center. Orthogonal crystal faces are colored blue and orange. (c–f) Polarized optical micrographs (POMs) of banded spherulites of coumarin with 20 wt % Damar gum, 2,5-bis(3-dodecyl-2-thienyl)thiazolo[5,4-*d*] (BDT), a charge transfer complex of triphenylene and 2,3,5,6-tetrafluoro-1,4-benzoquinone, and triisopropylsilylethynyl anthradithiophene (TIPS ADT) with 20 wt % medium density polyethylene (PE), respectively. Melting points (mp) and crystal space groups are also provided.

“Circular crystals” with concentric rhythmic contrast were first described by Brewster in 1853 (Figure 1a).^52,53^ Some of these were surely twisted (i.e., hippuric acid), albeit he did not recognize them as such. Wallerant in 1905–1907^23,54−58^ recognized that comparable objects were comprised of crystal fibers that smoothly rotated as they grew from a central nucleus (Figure 1b). The rhythmic repetition of interference colors between crossed polarizers is characteristic of helicoidal twisting, which is distinct from nonbanded spherulites comprised of straight crystals. Pitches, or the spacing between like-colored interference bands, typically ranged from the submicrometer to millimeter length scale. In 1929, Bernauer estimated that more than one-quarter of all small-molecule melts can be induced to grow as banded spherulites.^23^ His research was interrupted by the Second World War.^26^ Crystal twisting was rediscovered in the second half of the 20th century in poly(ethylene) and other synthetic polymers.^59−68^ The work on small molecules was largely forgotten, as evidenced by the widespread assumption that helicoidal morphologies are exclusive to macromolecules.^59,60,69−72^

Over the past 12 years, our research team and others have detailed crystal twisting in more than 100 small-molecule compounds,^22^ including hippuric acid,^27,31^ resorcinol,^73^ potassium dichromate,^19,26^ tetraphenyl lead,^26^ testosterone propionate,^32^d-mannitol,^24,74,75^ benzamide,^76^ paracetamol,^77^ ROY,^78^ and aspirin,^79^ among many others. Crystal twisting is agnostic to molecular and crystal symmetry. Figure 1c–f displays four examples of banded spherulites of molecular compounds and complexes that crystallize in a variety of space groups. While the compounds and crystals that comprise banded spherulite films need not be chiral, twisting necessarily imparts chirality. Helicoidal crystals can be right- or left-handed, with spherulites sometimes bisected into two domains comprising heterochiral helicoids. Banded spherulites exhibit optical activity, albeit this is not natural optical activity^80^ of homogeneous media but instead arises from the splay sense of crystals perpendicular to the light propagation direction.^81^

Additives,^58^ including Canada balsam,^23^ Damar gum,^22^ poly(vinylpyrrolidone),^24,29^ poly(ethylene),^82^ and abietic acid^83^ are sometimes incorporated at 5–30 wt % to induce twisting. These additives suppress nucleation at large undercoolings and increase melt viscosity, which promotes the crystallization of long, needlelike fibrils that have a greater propensity to twist than thicker crystals.^50^ Several mechanisms for twisting have been proposed, and it is unlikely that a single mechanism is responsible for spontaneous morphological deformations across such a broad spectrum of compounds, symmetries, and crystallization conditions.^50^ Arguably the most general mechanism arises from the fact that oddly shaped molecules conform to the rules of classical crystallography.^84−86^ Other mechanisms were reviewed previously.^50^ Uncompensated surface stresses may be especially important in polymers with very thin lamellae.^68^

### Twisting in Organic Semiconductor Crystals

This perspective focuses on crystal twisting in a subset of molecular compounds that are semiconducting, i.e. those with relatively small energy differences (∼4–5 eV or smaller) between the highest occupied molecular orbital (HOMO) and lowest unoccupied molecular orbital (LUMO). Optical and electrical properties, including absorbance, photoluminescence, and conductivity, are strongly dependent on the HOMO–LUMO gap and intermolecular interactions. The former is determined by the molecular structure, while the latter depends on how the molecules pack in the solid state. Charge conduction in particular occurs through intermolecular overlap of delocalized π-orbitals, with the extent of π-orbital overlap being characterized by the charge transfer integral, *J*.^87^*J* depends on the crystallographic direction, leading to ⟨*hkl*⟩-dependent charge mobilities in organic semiconductor crystals that can vary over several orders of magnitude.^88^ Twisting on the mesoscale has a small effect on *J* itself—a pitch, *P*, of 1 μm corresponding to a 180° rotation about the growth direction translates to a <0.1° rotation between adjacent molecules in a crystal. Indeed, crystal lattice parameters of straight and twisted crystals are nearly indistinguishable from one another.^89^ Instead, twisting introduces (1) periodicity in material properties, including charge mobility, on the micrometer length scale, as crystal orientations continuously rotate about the growth directions and (2) chirality through the twist sense.

Twisting has been reported for a limited number of organic semiconductors to date. Poly(3-butylthiophene) (P3BT), for example, forms banded spherulites when crystallized in the presence of poly(ethylene) (PE), but optoelectronic properties were not investigated.^90^ Chiral diketopyrrolopyrroles were recently reported to form twisted single crystals from solution.^91^ The twist sense was determined by the molecular chirality, with enantiomorphous twisting in opposite directions. These crystals exhibit circular dichroism and circularly polarized luminescence, making them promising candidates for chiroptoelectronics. A thieno[3,2-*b*]thiophene oligomer was also previously reported to form banded spherulites with a twisting pitch of 25 μm when crystallized from the melt, but the effect of crystal twisting on optoelectronic properties was not examined.^92^ Optically active twisted liquid crystal phases of an achiral isoindigo-bithiophene-based copolymer were also recently discovered.^93^ Helical copolymers exhibited more efficient intermolecular energy transfer compared to nonhelical polymers, which may lead to improved solar conversion efficiencies in organic solar cells, albeit the mechanism underlying the improvement is uncertain.

**Figure 2:**
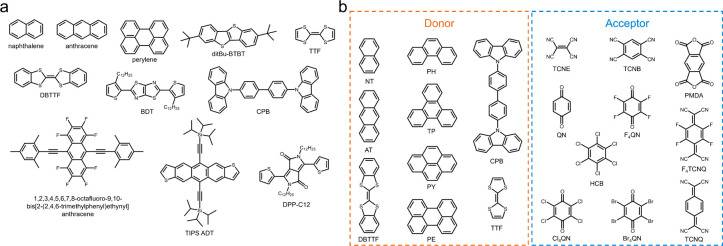
Molecular structures of (a) monocomponent organic semiconductors and (b) donors and acceptors in charge transfer complexes that form banded spherulites. Abbreviations: ditBu-BTBT, di*tert*-butyl [1] benzothieno[3,2-*b*][1]1benzothiophene; TTF, tetrathiafulvalene; DBTTF, dibenzotetrathiafulvalene; BDT, 2,5-bis(3-dodecyl-2-thienyl)-thiazolo[5,4-*d*]thiazole; CPB, 4,4′-bis(*N*-carbazolyl)-1,1′-biphenyl; TIPS, triisopropylsilylethynyl; ADT, anthradithiophene; DPP-C12, 2,5-didodecyl-3,6-di(thiophen-2-yl)-2,5-dihydropyrrolo[3,4-*c*]pyrrole-1,4-dione; NT, naphthalene; AT, anthracene; PH, phenanthrene; TP, triphenylene; PY, pyrene; PE, perylene; TCNE, tetracyanoethylene; TCNB, tetracyanobenzene; PMDA, 1*H*,3*H*-benzo[1,2-c:4,5-c′]difuran-1,3,5,7-tetraone; QN, quinoline; F_4_QN, tetrafluoroquinoline; HCB, hexachlorobenzene; F_4_TCNQ, tetrafluorotetracyanoquinodimethane; Cl_4_QN, tetrachloroquinoline; Br_4_QN, tetrabromoquinoline; TCNQ, tetracyanoquinodimethane. Adapted with permission from ref (89). Copyright 2022, American Chemical Society.

Over the past several years, we have identified at least 11 organic semiconductors that readily form banded spherulites from the melt (Figure 2a) in addition to a library of binary charge transfer complexes (CTCs) prepared from 9 donors and 10 acceptors (Figure 2b).^89^ The fraction of CTCs that twist, 23/41 = 56%, is significantly higher than those of arbitrarily selected single-component molecular crystals: 130/480 = 27% reported by Bernauer^23^ and 48/155 = 31% in our recent study.^22,94^ In this perspective, we highlight our recent findings on band-dependent properties of rhythmically twisted organic semiconductor spherulites and emergent properties related to twist-induced chirality. An outlook on the future of twisted organic semiconductor crystals is provided.

## Twist-Patterned Properties

**Figure 3:**
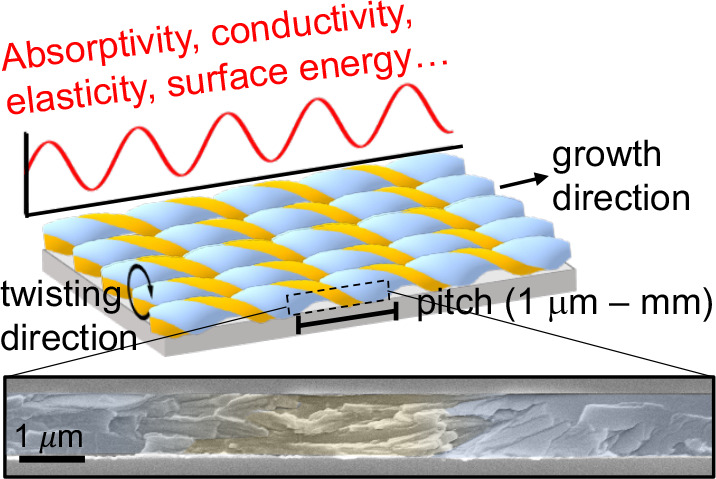
Illustration of orange and blue faces of the helicoidal fibrils exhibiting different magnitudes of ⟨*hkl*⟩-dependent material properties and a cross-sectional SEM image of a TIPS ADT banded spherulite film with face-on and edge-on orientations colored blue and orange, respectively.

Rhythmic crystal twisting results in a continuous rotation of crystal faces exposed at the film surface. Films comprise bundles of helicoidal fibrils that grow radially outward from the spherulitic nucleus and twist about the growth direction in concert with one another. The fibrils typically exhibit wide (H) and narrow (h) faces, colored blue and orange, respectively, in Figure 3. “Face-on” and “edge-on” crystal orientations refer to presentations of wide and narrow faces of lamellae that are alternately parallel to the substrate surface, respectively. *P* scales with *h* according to a power law, *P* = const·*h*^*n*^, where *n* ranges from 1.8 to 5.4 for small-molecule banded spherulites.^24^ The exponent reflects a balance of twisting and untwisting as well as elastic and plastic deformations.^31,95^ A cross-sectional SEM of a triisopropylsilylethynyl anthradithiophene (TIPS ADT) banded spherulite collected along the growth direction displays distinct regions of face-on and edge-on crystal orientations in bundles of helicoidal fibrils that twist cooperatively (Figure 3). All ⟨*hkl*⟩-dependent material properties are patterned into banded spherulite films with frequencies determined by *P*. This section provides an overview of properties that follow this pattern, namely linear dichroism, linear birefringence, fluorescence, charge mobility, solubility, and reactivity.

### Band-Dependent Linear Anisotropies

Colored, anisotropic, dissymmetric, heterogeneous (along the light path) crystals are a joy for polarimetry. Banded spherulites are arresting to look at and the modulation of optical properties is obvious.

The linear polarization properties that one would ideally like to extract from such samples are given in Table 1. In the definitions therein *n* and κ represent refractive indices and absorption coefficients, respectively, the components of the complex refractive index, *n′ = n – iκ*. Subscripts in the formulas represent the angle of linearly polarized light (0, 90, 45, −45°), or the handedness of circularly polarized light (L, R). The tilde indicates isotropic averages.

**Table 1:** Fundamental Linear Optical Polarization Properties

polarization properties	symbol	definition
total refractive index	*ñ*	*n*_0_ + *n*_90_
total absorption	–*κ̃*	κ_0_ + κ_90_
horizontal linear birefringence	LB	*n*_0_ – *n*_90_
horizontal linear dichroism	–LD	κ_0_ – κ_90_
45° linear birefringence	LB′	*n*_45_ – *n*_–45_
45° linear dichroism	–LD′	κ_45_ – κ_–45_
circular birefringence	CB	*n*_L_ – *n*_R_
circular dichroism	–CD	*κ*_L_ – *κ*_R_

**Figure 4:**
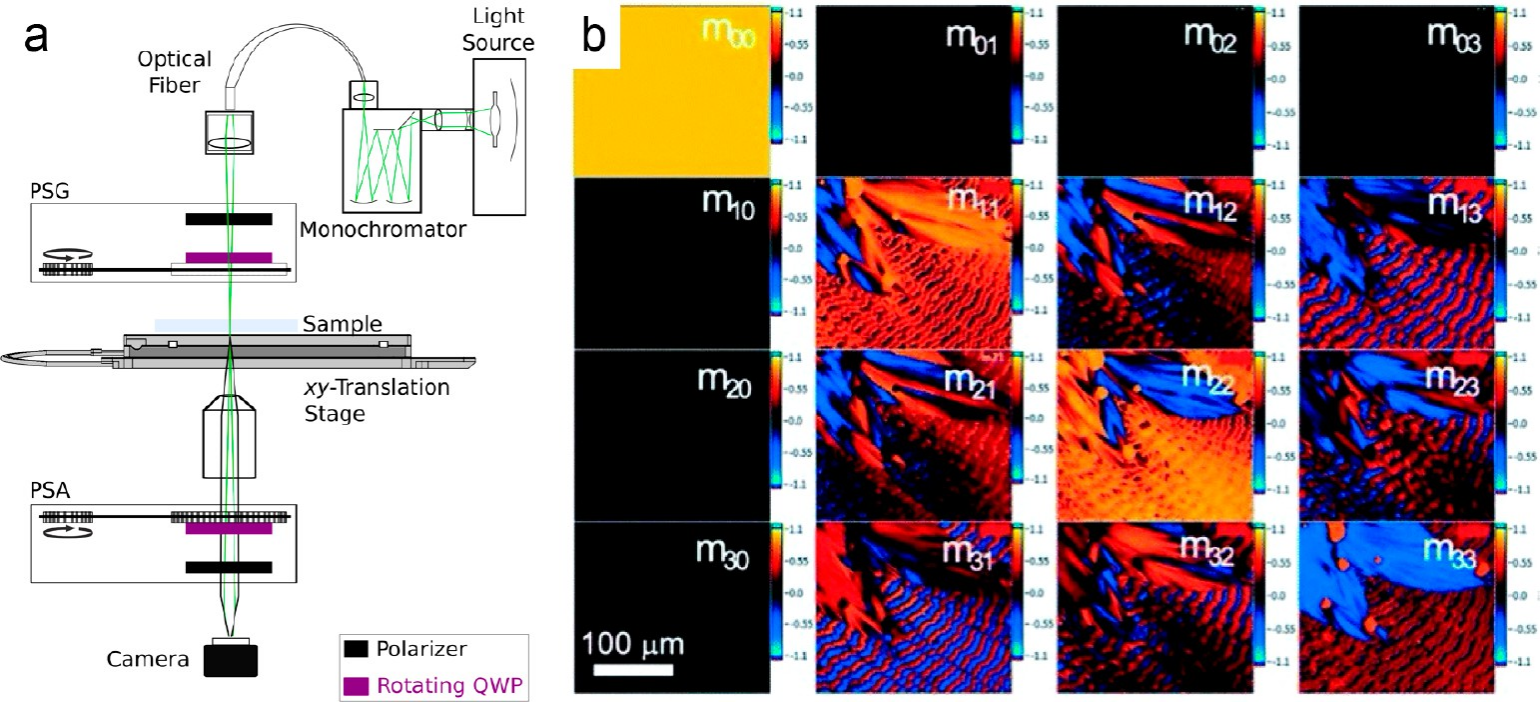
(a) Mueller matrix microscope setup. Abbreviations: PSG, polarization state generator; PSA, polarization state analyzer; QWP, quarter waveplate.^98^ (b) Mueller matrix of a TTF banded spherulite measured at 500 nm. Adapted with permission from ref (99). Copyright 2022, Wiley-VCH.

These quantities can be extracted by so-called complete polarimetry.^96^ A complete polarimeter delivers all the 16 elements of the [4 × 4] polarization transfer or Mueller^97^ matrix. A home-built Mueller matrix microscope using a commercial Zeiss base (Zeiss Z1 Observer) is shown in Figure 4a.^98,171^ The instrument is composed of five main parts: a light source, a polarization state generator (PSG), a sample stage, a polarization state analyzer (PSA), and an imaging system containing a camera and lenses. A spectroscopic light source constructed from a white LED coupled to a monochromator with adjustable grating is applied. A light guide brings the source to the microscope. The PSG and PSA each contain a manually adjustable polarizer and a motorized continuously rotating retarder (quarter waveplate) to produce and subsequently analyze the polarization state of light.

**Figure 5:**
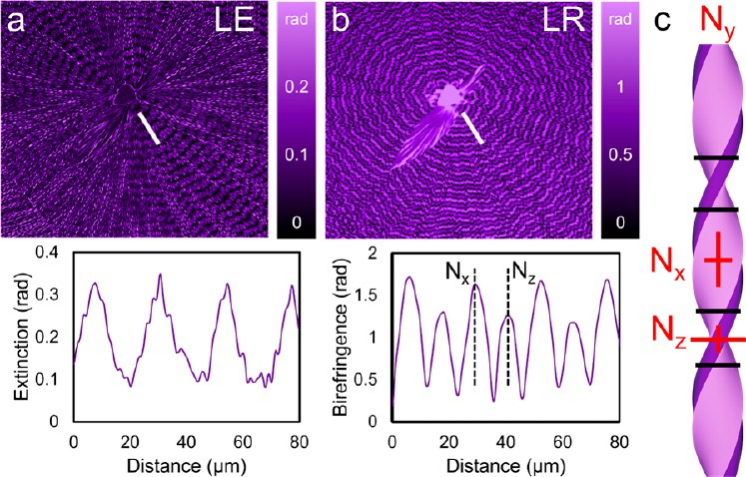
(a, b) LE and LR maps of a TTF banded spherulite generated by Mueller matrix microscopy. Line profiles extracted from the images along the white lines are also provided. (c) Illustration of a helicoidal fibril in which the refractive indices along directions perpendicular to the incident light direction are indicated.

Figure 5 displays Mueller matrix maps of the linear extinction (LE) and linear retardance (LR) signals of a TTF banded spherulite.^99^ Dichroism and birefringence are intrinsic properties whereas extinction and retardance are extrinsic properties that depend on path length. Extinction and retardance are also more phenomenological and mechanistically agnostic. We previously determined that the radial growth direction is along ⟨010⟩, with alternating bands corresponding to the (100) and (001) planes oriented parallel to the substrate surface. Periodic oscillations in both LE and LR emanating radially from the spherulite nucleus are observed. The LE signal oscillates between 0.1 and 0.3 rad, and the LR signal has alternative maxima of 1.7 and 1.3 rad. These oscillations are due to different absorbances and refractive indices along different directions in TTF crystals. Figure 5c displays an illustration of a helicoidal fibril in which the refractive index along the long axis of the fibril assigned as *N*_*y*_. *N*_*x*_ and *N*_*z*_ are orthogonal to *N*_*y*_ and correspond to the wide and narrow faces of the fibril, respectively. The birefringence along the fibril long axis oscillates between values of |*N*_*y*_ – *N*_*x*_| and |*N*_*y*_ – *N*_*z*_| as it twists, resulting in concentric bands of LR signal emanating from the spherulite center.

**Figure 6:**
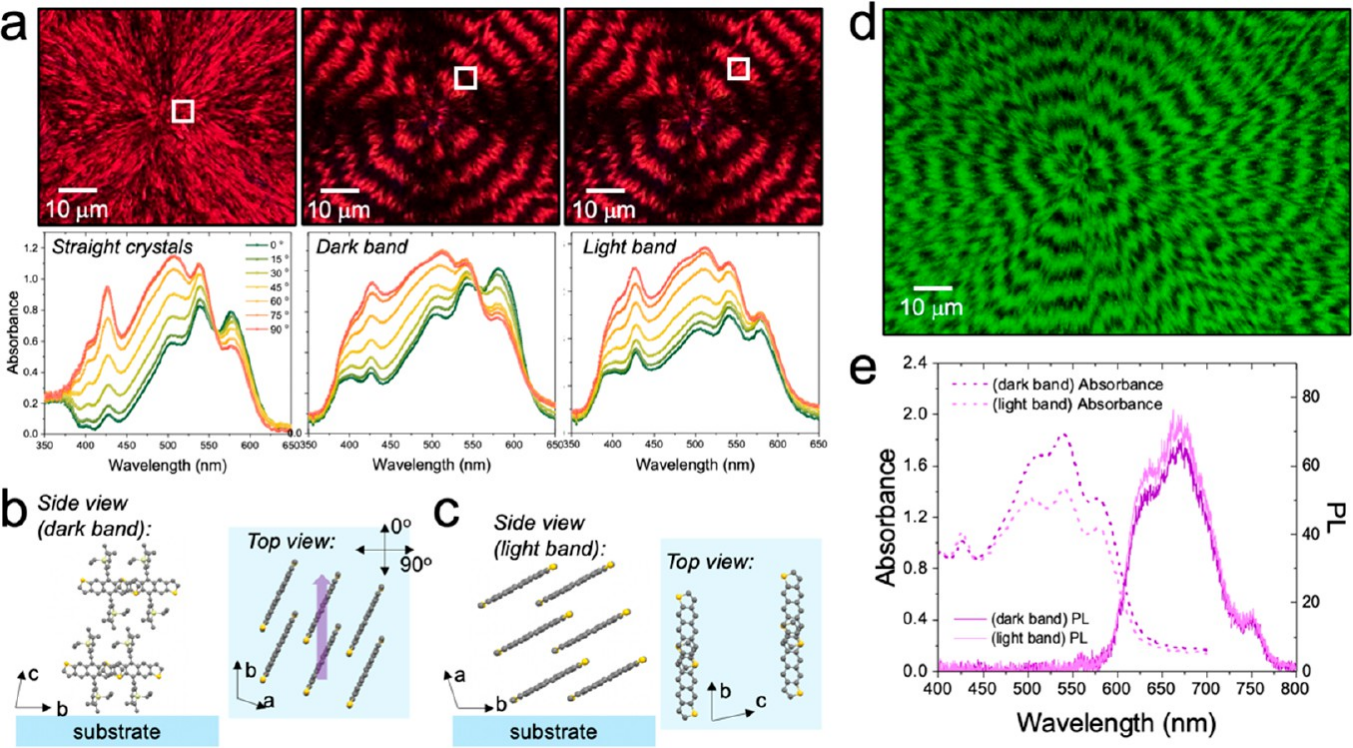
(a) Optical micrographs of nonbanded and banded TIPS ADT spherulites between crossed polarizers and corresponding polarization-angle-dependent absorption spectra collected in the regions highlighted by white squares. (b, c) Illustration of molecular orientations in dark and light bands, respectively. (d) Fluorescence image of a TIPS ADT banded spherulite (λ_ex_ = 546 nm). (e) Unpolarized absorption and photoluminescencespectra collected on dark and light bands of a TIPS ADT banded spherulite. Adapted with permission from ref (82). Copyright 2023, Wiley-VCH.

Wavelength-dependent absorption spectra of twisted organic semiconductor crystals also exhibit band dependencies. Figure 6a displays POMs and corresponding polarization-angle-dependent absorption spectra for nonbanded and banded TIPS ADT spherulites comprising straight and twisted crystals, respectively.^82^ A microspectrophotometer (CRAIC Technologies) mounted on an optical microscope was used to collect localized spectra in the 5 × 5 μm regions highlighted with white squares. The lowest energy peak at 577 nm, corresponding to the π–π* transition, exhibited a strong polarization angle dependence for both straight crystals and twisted crystals in dark bands of spherulites for which the ⟨001⟩ crystal direction is perpendicular to the substrate surface. In this orientation, maximum light absorption occurs when the polarization angle of light aligns with the π-stack direction in TIPS ADT crystals, indicated by the purple arrow in Figure 6b. The polarization angle dependence of this peak for twisted crystals in the light band, on the other hand, is weak, while other transitions exhibit a stronger polarization angle dependence compared to those in dark bands where crystals are oriented with the ⟨100⟩ direction perpendicular to the substrate (Figure 6c). Overall, aligning different crystal orientations with the direction of incoming light through crystal twisting presents a strategy to increase light absorption across the solar spectrum in organic semiconductor films. Such twist-induced improvements are expected to be particularly significant for films of pleochroic crystals, such as TIPS pyranthrene crystals that appear red or green depending on their orientation.^100^

### Band-Dependent Photoluminescence

Many organic semiconductors exhibit photoluminescence and electroluminescence in which light or electrically excited photons relax back to the ground state through the emission of a photon in the visible light range. Figure 6d displays a fluorescence micrograph of a banded TIPS ADT spherulite (λ_ex_ = 546 nm) and the corresponding band-dependent absorption and PL spectra. Like the pattern observed in the birefringence and absorption signals, the fluorescence exhibits periodic oscillations in intensity commensurate with the twisting pitch. Bands that absorb more strongly (dark bands with a face-on orientation) exhibit slightly weaker fluorescence compared to bands with lower absorption (light bands with an edge-on orientation; Figure 6e). This modulated fluorescence may be due to anisotropic light emission along different crystallographic directions. Waveguiding may also play a role.^101^ Edge-on orientations that expose narrow faces to the film surface likely exhibit lower waveguiding efficiency, and thus higher photoluminescence signal. BDT and di-*tert*-butyl[1]benzothieno[3,2-*b*]benzothiophene (ditBu-BTBT) banded spherulites also exhibit similar band-dependent photoluminescence.^102^

### Band-Dependent Conductivity

Charge transport anisotropy along different crystallographic directions has been quantified for a number of organic semiconductors, including rubrene,^103^ triisopropylsilylethynyl pentacene (TIPS pentacene),^88^ and TIPS pyranthrene.^7^ These crystals often adopt a preferred out-of-plane orientation in solution- and melt-processed films with the π-stack direction parallel to the substrate surface. In banded spherulites, the in-plane radial growth direction typically corresponds to the π-stack direction, while out-of-plane orientations continuously rotate between low- and high-surface energy faces perpendicular to the π-plane. Interestingly, transistors comprising banded spherulite active layers exhibit higher mobilities than their nonbanded spherulite counterparts for at least five different molecular semiconductors and charge transfer complexes, including pyrene-tetracyanoethylene (PyT), phenanthrene-tetracyanoethylene (PhT),^89^ and TTF.^99^ Increasing charge mobility with decreasing twisting pitch was observed for transistors comprising banded spherulites of BDT.^102^ BDT transistor hole mobilities increased from 0.5 × 10^–3^ to 1.6 × 10^–3^ cm^2^ V^–1^ s^–1^ for twisting pitches decreasing from *P* = 160 μm to *P* = 35 μm, respectively, when the spherulitic growth direction was parallel to the current flow direction between the source and drain. These improvements in hole mobilities with decreasing *P* in the banded spherulite active layer were primarily attributed to differences in film morphology. Molecular crystals attached to a glass substrate often crack upon cooling. Nonbanded spherulite films exhibited few cracks, but the fissures are comparatively large. Banded spherulite films typically have a larger number of small cracks. Electric potential distributions in films were simulated by binarizing scanning electron micrographs upon which were superimposed square lattices of resistors, proportional to the conductivity in anisotropy, and interrupted by the cracks. Film conductance values calculated from electric potential distributions followed the same trend as experimental measurements, with conductance increasing with decreasing pitch.

**Figure 7:**
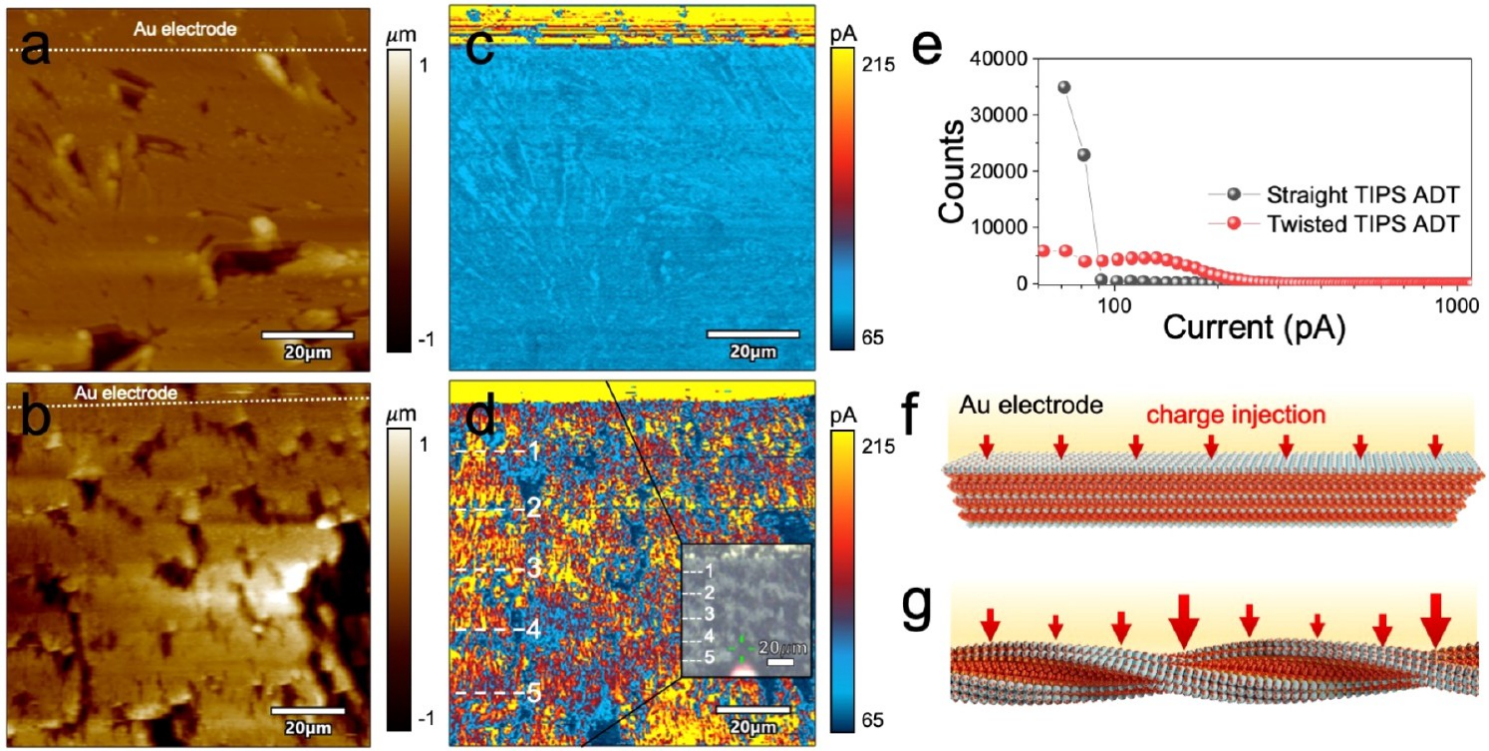
AFM height images (a, b) collected in contact mode and corresponding lateral current mapping images of (c, d) films of straight and twisted crystals, respectively. Optical micrograph of the scanned region is provided as an inset in (d), with corresponding bands numbered in the c-AFM map. (e) Histograms that tabulate the counts of current level of the films and twisted. Schematic illustration of (f) straight and (g) twisted TIPS ADT crystals at the film/electrode interface. Red arrows qualitatively illustrate the magnitude of charge injection at different locations along the crystal. Reproduced with permission from ref (82). Copyright 2023, Wiley-VCH.

Following this work, we used conductive atomic force microscopy (AFM) to measure band-dependent conductivity in TIPS ADT spherulite films. Unlike BDT transistors, TIPS ADT transistors exhibit measurable current levels when no gate bias is applied (i.e., they are leaky), allowing conductivity maps to be collected using two-terminal devices. For these measurements, gold electrodes were thermally evaporated onto TIPS ADT films of both nonbanded and banded spherulites. Current was measured laterally across the film surfaces from the gold electrode to a conductive AFM tip. Figure 7 displays height maps and corresponding current maps collected at an applied bias of 10 V for nonbanded (a, c) and banded (b, d) TIPS ADT spherulitic films. The current level for the banded spherulite film was two times higher than those measured for the nonbanded spherulite film (Figure 7e). Periodic oscillations in current levels were also observed in the current map of the banded spherulite, with the frequency corresponding to the pitch. We expect current values to be low in TIPS ADT straight crystals because these crystals are oriented with continuous layers of insulating silyl groups parallel to the substrate surface that act as barriers to charge injection and extraction (Figure 7f). TIPS ADT twisted crystals, on the other hand, also expose the anthradithiophene core to the film surface in alternating bands, facilitating charge injection and extraction (Figure 7g). Improved conductivity in alternating bands served to increase the conductivity of TIPS ADT films overall; photocurrents were threefold larger.

### Solubility

**Figure 8:**
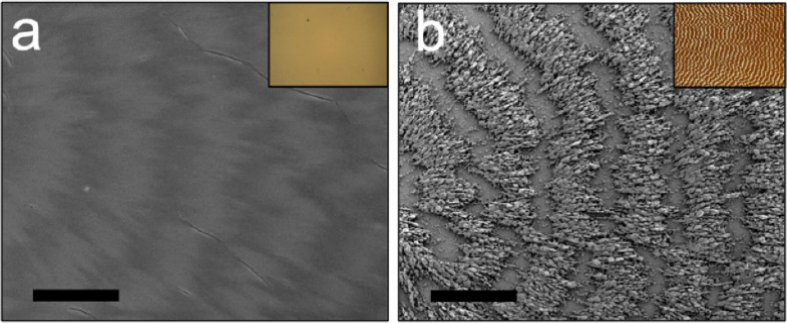
SEMs of a TTF banded spherulite (a) before and (b) after 24 h exposure to methanol solvent vapor. Bright-field optical micrographs are provided as insets. Adapted with permission from ref (117). Copyright 2023, American Chemical Society.

Integrating organic semiconductor films into multicomponent optoelectronic devices will require spatial patterning of the films to connect components and reduce current leakage.^104^ Several strategies include confining crystallization within polymer molds,^105−107^ patterning self-assembled monolayers on substrates to promote preferential film wetting during organic semiconductor deposition^108,109^ or to control organic semiconductor crystallization rates,^110^ as well as rhythmic precipitation.^111^ Twisting also patterns organic semiconductor films on the micrometer to millimeter length scale because different crystal faces exhibit anisotropic surface energies and morphologies. As crystal fibers rotate about the growth direction, the narrow, high-surface-energy faces and wide, low-surface-energy faces of the ribbon-like fibrils are alternately exposed at the surface of the film.^24,38,64,79,112−114^ Crystal dissolution rates are face-dependent,^115,116^ and the narrow, high-surface-energy bands in twisted crystals are expected to dissolve more quickly than the wide, low-surface-energy bands. TTF banded spherulites, for example, exhibit band-dependent dissolution in the presence of methanol.^117^Figure 8 displays time-lapse SEM and optical micrographs of a TTF banded spherulite film exposed to methanol vapor for a period of 0–24 h. Selective dissolution and recrystallization occurred on dark and bright interference bands, respectively. After 24 h of methanol vapor exposure, TTF crystallites organized into isolated, polycrystalline ridges with spacings determined by the as-grown pitch. An epitaxial relationship was observed between crystal orientation in the original banded spherulite film and the recrystallized ridges along and perpendicular to the growth direction. Because pitches can be varied from the submicrometer to millimeter length scale depending on crystallization temperature, additive concentration, and other factors, ridge widths and spacings can be tuned accordingly.

### Reactivity

**Figure 9:**
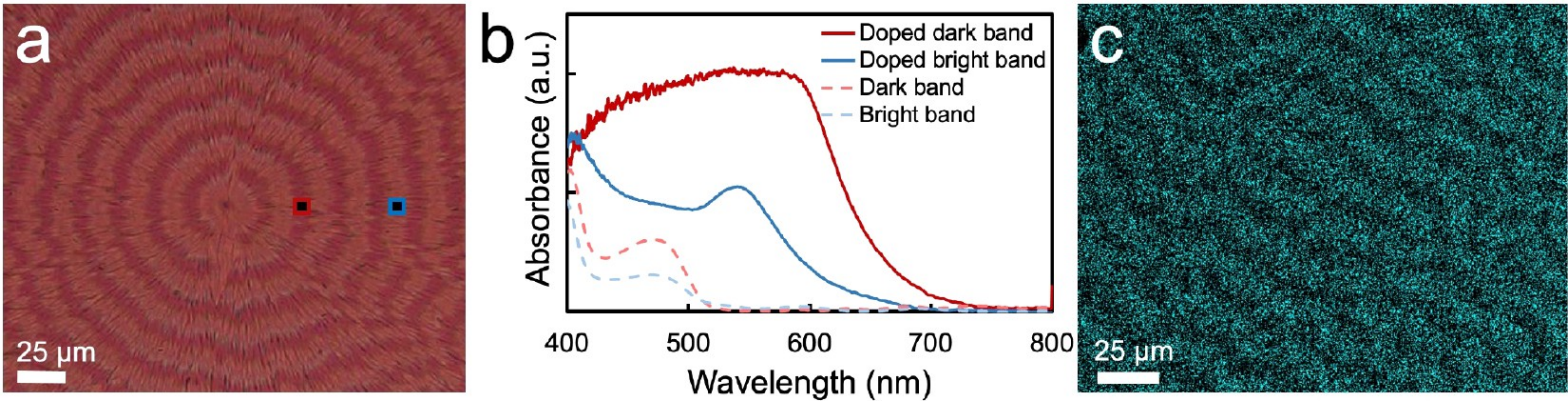
(a) Optical micrograph of a banded TTF spherulite exposed to iodine vapor for 5 min. (b) Band-dependent absorbance spectra of the film in a). (c) Energy dispersive X-ray spectroscopy map of iodine in a TTF banded spherulite after 5 min of exposure to iodine vapor.

Chemical reactivity is another material property that depends on the crystallographic face exposed at film surfaces. For example, iodine doped thin films of 7,7′-(4,4-bis(2-ethylhexyl)-4*H*-silolo[3,2-*b*:4,5-*b*′]dithiophene-2,6-diyl) bis(6-fluoro-4-(5′-hexyl-[2,2′-bithiophen]-5-yl)benzo[*c*]thiadiazole were found to result in preferential edge-on alignment.^118^ TTF can form various salts with iodine or polyiodides that are more conductive than undoped TTF.^119−123^ With increasing iodine vapor exposure time, yellow TTF films became dark magenta and the conductivity increased by 6 orders of magnitude. Energy dispersive spectroscopy (EDS) showed that the iodine incorporation rate varied on alternating bands at early iodine vapor exposure times (Figure 9). Specifically, iodine preferentially reacted with TTF crystals in dark false color bands corresponding to the edge-on orientation. Because conductivity increases with increasing iodine doping, band-selective reactivity is expected to result in band-selective conductivity.

## Emergent Optoelectronic Properties

Helicoids are chiral, leading to emergent properties.

### Circular Dichroism and Birefringence

Crystal twisting obviates symmetry operations of the second kind. Individual crystallites in banded spherulites can be homochiral or heterochiral. For centrosymmetric molecular crystals that crystallize as banded spherulites, twist sense is determined during spherulite nucleation.^102^ Enantiopolar faces of the first nucleus become unstable, growing in opposite hemispheres with opposing twist senses. Such banded spherulites may be bisected into heterochiral domains, the area of which can be influenced with chiral additives.^30,83^ Twist sense in resorcinol crystals, for example, can be selected with d- or l-tartaric acid additive.^73^ Crystal twisting may impart optical activity to achiral compounds and crystals and transform optoelectronic materials to *chir*optoelectronic materials.

**Figure 10:**
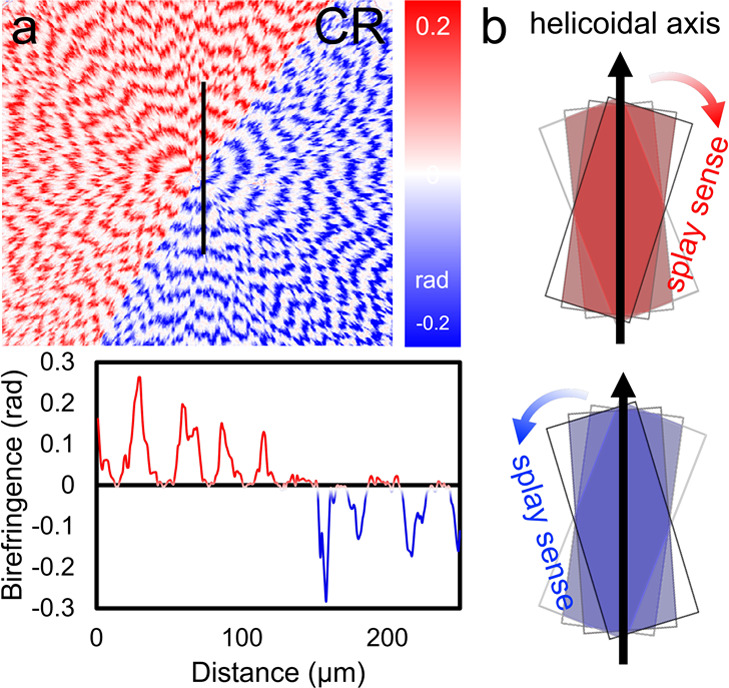
(a) CR map of a BDT banded spherulite generated by Mueller matrix microscopy. A baseline corrected line profile extracted from the image along the black line is also provided. (b) Illustration of splayed stacks of crystallites with opposing splay senses.

As described above, Mueller matrix imaging is complete polarimetry^124^ that delivers all the components of the differential polarization operator, including the circular extinction (CE) and circular retardance (CR). Figure 10 displays a CR map of a banded BDT spherulite. Concentric bands in the signals correspond to oscillating refractive indices with different crystallographic directions. Furthermore, the CR micrograph reveals that the spherulite is bisected into heterochiral semicircles, one dextrorotatory and the other levorotatory, corresponding to opposing twist senses.^125^ We stress that the signal is not natural optical activity but arises in the sense of splay of thin anisotropic lamellae, as displayed in Figure 10b.

A model describing the superimposition of twisted crystallites, causing misalignment on the *z* direction and resulting in circular birefringence, has been proposed.^81,126^ This mechanism is consistent with Reusch’s pile of misoriented mica plates, an early model of optical rotation.^127^ Small rotation between small anisotropic layers produce CB. Mueller matrices of each fiber (***M***_*k*_) of the *k*th (*k* = 1, 2, 3, ..., *N*) layer was built with the same linear birefringence (LB/*N*) in term of progressive rotation angle φ/(*N* – 1)
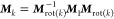
1where
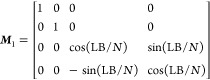
2and
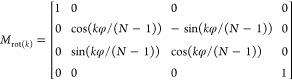
3

Thus, the total Mueller matrix can be calculated as
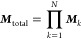
4

The total misalignment angle φ oscillates from zero for the edge-on and flat-on and becomes φ_max_ and −φ_max_ at middle points between, which can be expressed by
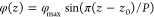
5where *P* is the twisting period and *z*_0_ is the position of the edge-on orientation. With this model, the circular birefringence of twisted crystals, including durene, d-mannitol, aspirin, and poly(3-hydroxy-butyrate), has been simulated and the results are consistent with Mueller matrix measurements.

CE in films can be used for the detection and emission of circularly polarized light (CPL). For CPL photodetectors comprising banded spherulite active layers, we expect photocurrent levels to depend on the (mis)match between the crystal twist sense and the CPL sense. Photons traveling through stacks of misoriented crystals in banded spherulites may also gain some circular polarization. These applications remain to be explored.

### Iridescence

When the pitch in banded spherulites approaches the wavelength of visible light, constructive and destructive interactions between photons can give rise to iridescence. Iridescence, or structural color, is a phenomenon caused by the selective reflection of certain wavelengths of visible light from a microstructure, resulting in beautiful rainbow-like displays of colors that change when viewed from different angles.^128^ In banded spherulites, this iridescence is caused by the interference of light waves encountering alternating bands of different refractive indices, *n*_A_ and *n*_B_. The wavelength of reflected light is given by the equation^128^
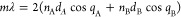
6where *d*_A_ and *d*_B_ are the thickness of layers A and B, respectively, corresponding to the width of each band in banded spherulites, and θ_A_ and θ_B_ are the angles of refraction in layers A and B, respectively. When *d*_A_ and *d*_*B*_, commensurate with the twisting pitch, *P*, approach the wavelength of visible light, iridescence can be observed. Iridescence has been previously observed in banded spherulites of poly(*p*-dioxanone),^129^ poly(ethylene adipate),^130^ poly(3-hydrobutyrate),^131^ and mannitol,^170^ among others.

## Future Directions

### Control over Twist Sense

One of the major challenges facing the use of growth-actuated twisted crystals in chiroptoelectronics is that achiral crystals can often twist both clockwise or counterclockwise about the growth direction. As displayed in Figure 10, banded spherulites are typically bisected into domains with opposing twist senses, which are determined during spherulite nucleation. In some cases, the ratio of clockwise (CW) to counterclockwise (CCW) domain areas can be biased by incorporating chiral additives,^73,83^ but isolating a single twist sense is generally not possible for achiral crystals in banded spherulites grown from the melt. For banded spherulites with heterochiral twists, the net interaction with circularly polarized light will be zero. Controlling the twist sense in banded spherulites is thus crucial for chiroptoelectronic applications that discriminately absorb, detect, and/or emit either left or right circularly polarized light.

**Figure 11:**
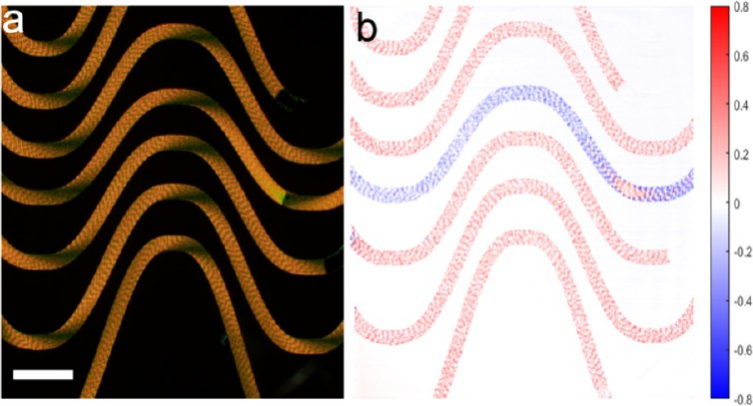
(a) Optical micrograph of BDT crystallized in the wavy channels of a PDMS mold between crossed polarizers and (b) corresponding map of the CR. Adapted with permission from ref (125). Copyright 2023, Wiley-VCH.

One promising strategy to overcome this limitation is to use polymer molds with prescribed geometries to spatially isolate bundles of homochiral twisted crystals.^125^Figure 11a displays BDT twisted crystals collimated in curved 100 μm wide, 5 μm tall channels in poly(dimethylsiloxane) (PDMS) molds between crossed polarizers. BDT was introduced into the channels in the melt phase through capillary forces and subsequently crystallized through rapid cooling below the melting point. The twisting bands were collimated in the channels as crystallization was confined in a single direction. The MMI map of the CR signal revealed that of the seven channels imaged, six of them exhibited positive CR that persisted throughout the entire channel. While it was not possible to dictate the twist sense *a priori*, homochiral helicoidal fibrils were isolable.

Spatially isolating the twist sense will enable the measurement of enantiomorph-dependent processes, such as magnetochiral anisotropy. This phenomenon manifests as anisotropic resistivity, *R*, through a material depending on the direction of an applied magnetic field. It has been previously demonstrated that a small internal magnetic field is generated when a voltage is applied parallel to the axis of a helicoidal conductive crystal, similar to a solenoid. The conductivity is maximized when an external magnetic field aligns with the internally generated field and minimized when the external field opposes the internal one,^132−139^ following the equation^139^
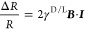
7where γ^D/L^ represents the magnetochiral anisotropy factor between right and left handed twists, ***B*** is the magnetic field, and ***I*** is the current. Electric magnetochiral anisotropy has been observed in twisted bismuth wires,^132^ crystals of a chiral TTF derivative,^133^ and carbon nanotubes,^140^ among other structures. We expect twisted organic semiconducting and conducting crystals will likewise exhibit electric magnetochiral anisotropy.

### Driving Twisting Pitches Smaller

Pitches, *P*, in banded spherulites grown from the melt typically range from the micrometer to hundreds of micrometers length scales. A 10 μm pitch corresponding to a 180° rotation in crystal orientation translates to an ∼0.01° rotation between adjacent molecules. DFT calculations to simulate charge transport through charge transfer complex crystals predict only modest improvements in hole and electron mobilities upon crystal twisting due to small changes in the charge transfer integral, *J*, between adjacent molecules.^89^ We expect that the twist rate leads to larger differences in intermolecular interactions compared to those in straight crystals, which will in turn affect material properties, including conductivity, absorptivity, and photoluminescence, among others. In comparison, chiral bowtie particles with tunable sizes and twist intensities exhibited progressive shifts in CD spectra.^141^ It is also possible that smaller twisting pitches will give rise to new phenomena related to chirality on the molecular length scale. In twisted-bilayer graphene, for example, the first magic angle between graphene sheets at which superconductivity is observed is 1.05°.^142,143^ Twisted crystals might manifest the chiral-induced spin selectivity (CISS) effect. The CISS effect, in which chiral compounds selectively conduct electrons depending on their spin state,^144^ has been observed in a number of different compounds, including chiral molecules,^145−148^ perovskites,^149,150^ metal–organic frameworks,^151,152^ and crystals with chiral superlattices.^153^

In general, *P* is positively correlated to the crystallization temperature—lower crystallization temperatures (i.e., larger supercoolings that increase the crystallization driving force) promote the growth of finer fibrils that can twist with higher frequency.^24,26,82,102^*P* typically reaches a minimum value at some intermediate supercooling, after which further supercooling does not affect *P*. In TIPS ADT banded spherulites, for example, a minimum *P* of 10 μm is observed across the range of 20–80 °C.^82^ We recently discovered the twisting pitch of d-mannitol banded spherulites in the presence of 15 wt % poly(vinylpyrrolidone) (PVP), an additive incorporated to induce crystal twisting, decreased from 27 μm under quiescent conditions to 8 μm when exposed to steady torsional shear at a rate of 100 s^–1^.^74^ The dependence of *P* on the shear rate is likely related to differences in PVP chain conformation under shear flow, which in turn affects its interactions with d-mannitol crystals. Applying large shear rates to compounds with small twisting pitches under quiescent conditions may drive *P* lower but will likely depend on the specific compound and additive.

### Accessing the Twist Axis

**Figure 12:**
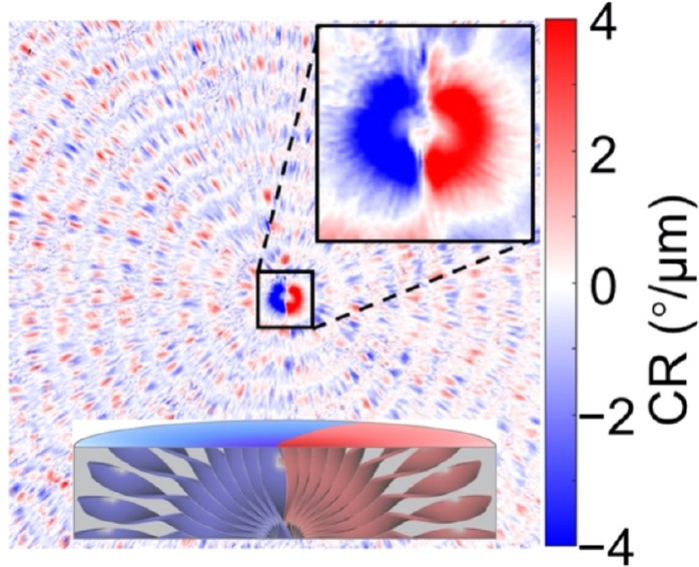
Circular retardance (CR) micrograph of poly(propylene fumarate) banded spherulites. The core is enlarged and inset (upper right). A scheme of heterochiral twists in the core is given at the bottom, viewed along a radius within the sample plane. The lateral dimension is 200 μm. Adapted with permission from ref (154). Copyright 2019, American Chemical Society.

In the 2D geometry of banded spherulite thin films, the twist axis is typically perpendicular to the direction of incident light. We have previously shown that the CR directed along twisted fibrils of polymers can be especially high at the very center of the polycrystalline ensemble where the fibrils are parallel to the wavevector of light (Figure 12).^154^ Promoting crystal growth perpendicular to the substrate surface is thus expected to amplify many of the emergent properties discussed in this perspective, including CR, CE, and CPL. One strategy for orienting the fibril growth direction perpendicular to the substrate surface is through the use of nanoconfining scaffolds.^105,155^ In nanoconfined spaces, such as the cylindrical nanopores of anodized aluminum oxide scaffolds, crystals tend to grow with the fast growth direction parallel to the long axis of the nanopore.^156,157^ We have previously demonstrated the scaffold-directed solution-phase crystallization of semiconducting triisopropylsilylethynyl pyranthrene,^158^ perylene^159,160^ and formamidinium lead iodide.^161^ Organic semiconductors infiltrated into anodized aluminum oxide scaffolds from the melt likewise preferentially orient with the fast growth direction parallel to the long axes of the confining pores.^162^ Helical filaments of compounds with a liquid crystal phase were recently grown in anodized aluminum oxide nanopores for which the twist axis is aligned parallel to the pore axis.^141,163,164^ We expect that growing twisted organic semiconductor crystals in nanoporous scaffolds, especially (semi)conducting scaffolds that can participate in optoelectronic processes,^161^ will enhance the differential absorption, emission, and detection of circularly polarized light.

## Conclusions

To date, we have established that (1) spontaneous crystal twisting is common in melt-processed organic semiconductor crystals, especially those comprising charge transfer complexes, (2) ⟨*hkl*⟩-dependent material properties are modulated by twisting, (3) transistors and photodetectors comprising twisted crystals in the active layer consistently outperform those with straight crystals, and (4) twisting generates optical activity even for centrosymmetric molecules and crystals. Still, many questions surrounding the microstructure of twisted crystal films and the mechanisms governing their formation remain to be answered. The majority of emergent chiroptoelectronic properties arising from twist-induced chirality have also yet to be explored. Spontaneous crystal twisting will make available the huge library of organic semiconductors developed over the past 50 years for chiroptoelectronic applications ranging from chiral sensing^165^ to tissue pathology detection and information storage.^166−169^ The ubiquity of twisting across material classes and processing methods offers the repurposing of hundreds, if not thousands, of centrosymmetric compounds for chiral applications.
